# Gas composition and pressure in the hypopharynx during high-flow oxygen therapy through a nasal cannula in healthy volunteers with different breathing patterns

**DOI:** 10.1186/s12871-025-03267-9

**Published:** 2025-08-23

**Authors:** Andrey I. Yaroshetskiy, Anna P. Krasnoshchekova, Fedor D. Tkachenko, Alina V. Rubashchenko, Daniil D. Zubarev, Vasiliy D. Konanykhin, Maxim I. Savelenok, Maxim M. Nosenko, Zamira M. Merzhoeva, Sergey N. Avdeev

**Affiliations:** Pulmonology Department, Sechenov First Moscow State Medical University (Sechenov University), 8/2, Trubetskaya Str, Moscow, 119991 Russia

**Keywords:** Airway pressure, High-flow nasal cannula, Oximetry, Capnography, Physiological effects

## Abstract

**Background:**

High-flow nasal cannula is widespread in patients with hypoxemic and hypercapnic respiratory failure, but physiological data concerning influence of the combination of breathing pattern, preset flow rate (PFR), and inspiratory oxygen fraction (F_D_O_2_) on end-expiratory pressure (EEP), capnogram, oxygram, and exhaled tidal volume (VTe) remains insufficient.

**Methods:**

The study included 20 healthy subjects with 12 combinations of PFR (30–60-80 L/min) and F_D_O_2_ (40–60-80–100%) multiplied by 4 breathing patterns: mouth closed (CM), mouth open (OM), and combination of the CM and OM with hyperpnea (HCM and HOM). Pressure, capnogram, oxygram were measured from hypopharyngeal catheter, VTe, and subject’s comfort were assessed.

**Results:**

Inspiratory oxygen fraction (FiO_2_) were close to F_D_O_2_ at the PFR of 30 L/min (CM), and 60 L/min (HCM). FiO_2_ during the OM and HOM were much less than F_D_O_2_, variable and unpredictable. PFR of 60 L/min was sufficient to keep FiO_2_ close to F_D_O_2_ during the CM and HCM. End-expiratory carbon dioxide (F_E_CO_2_) decreased with an increase in the PFR and F_D_O_2_, reaching 1.4 (1.1–1.7)% at F_D_O_2_ 100% and PFR of 80 L/min. EEP had grown a lot with the PFR increase and were highly variable reaching 11.1 (7.7–14.8) cmH_2_O at the PFR of 80 L/min. VTe at the PFR of 60 and 80 L/min were 948.0 (715.0–1204.8) and 948.0 (869.0–1422.0) ml, respectively. PFR of 60 L/min and 80 L/min were associated with discomfort.

**Conclusion:**

HCM, OM, and HOM in healthy subjects decreased FiO_2_ and F_E_CO_2_ (more pronounced during OM and HOM). HFNC within the CM and HCM provided flow-dependent CPAP-effects over a wide range and could be associated with lung hyperinflation. An excessive PFR led to discomfort.

**Trial registration:**

ClinicalTrials.gov identifier: NCT06189716, registered on 19/12/2023.

**Supplementary Information:**

The online version contains supplementary material available at 10.1186/s12871-025-03267-9.

## Background

A high-flow nasal cannula (HFNC) is a promising tool for patients with respiratory failure that uses a combination of a continuously blended flow of air and oxygen delivered through a nasal cannula at preset flow rates close to or higher than the patient’s mean inspiratory flow [1]. Flow from the HFNC device is warmed and humidified according to physiological levels inside the trachea, bronchi, and even alveoli [2]. HFNCs are currently widely used for patients with hypoxemic and hypercapnic acute respiratory failure (ARF), including severe pneumonia, COVID-19, and COPD [3, 4]. The physiological effects of the HFNC are realized through more accurate inspiratory oxygen fraction (FiO_2_) titration [5–7], improved carbon dioxide (CO_2_) clearance [8–10], an elevated expiratory pressure (EEP) with an increase in the end-expiratory lung volume [11, 12], and better tolerance than noninvasive ventilation (NIV) [13].

Despite these effects and the clinical data concerning the efficacy of HFNCs, real O_2_ and CO_2_ fractions and end-expiratory pressure (EEP) close to the trachea are not well established, nor are data on the influence of different breathing patterns on these values [3, 4]. Changes in gas composition, expiratory pressure, and inspiratory/expiratory time with changes in breathing patterns could be of utmost importance because of their influences on the clinical efficacy and local or general side effects of HFNC therapy. For example, an increase in a subject’s minute ventilation from normal to 20 L/min during low-flow oxygen therapy through a facial mask (or nasal cannula) may decrease the FiO_2_ from 100 to almost 30%, thus greatly reducing the usefulness of oxygen therapy in patients with hypoxemia [14, 15].

Optimal combinations of the preset flow rate and preset oxygen fraction (F_D_O_2_) in patients with different breathing patterns are urgently needed to improve clinical efficacy and decrease the possible side effects of HFNCs. We performed our study to clarify these important points using simultaneous pressure, respiratory gases close to the trachea (in the hypopharynx), and tidal volume measurements in healthy volunteers with different breathing patterns.

## Methods

### Study design

The study was approved by the Institutional Ethics Committee (reference number: 25–23, date of approval 14/12/2023) and registered on ClinicalTrials.gov (NCT06189716). Written informed consent was obtained from all healthy volunteers enrolled in the study.

We recruited healthy volunteers (students, residents, and anaesthesiologists from Sechenov University) through oral advertisements and announcements in local social networks. The participants were not compensated. We recruited only nonsmokers or mild smokers (smoking index < 200, where the smoking index = cigarettes per day * years of smoking). Volunteers fasted for no less than 3 h before the study. 

Volunteers were seated in a chair. We applied topical anaesthesia with a lidocaine spray in both nostrils, on the surface of the tongue, and the pharynx. After two minutes, a lubricated multiperforated 8 French catheter was inserted into the hypopharynx through a nostril until the tip of the catheter was below the root of the tongue. The appropriate position of the catheter was checked by visual inspection throughout the study (after each step). Before all the measurements, the volunteers remained seated for 5 min to calm their breathing. At baseline, we recorded the atmospheric pressure from the local weather station and measured peripheral oxygen saturation (SpO_2_), the inspiratory and expiratory fractions of oxygen (FiO_2_ and FeO_2_, respectively), the end-tidal carbon dioxide fraction (FeCO_2_), the respiratory rate (RR), and the inspiratory and expiratory times (Ti and Te, respectively) using a side-stream multigas analyser (MPR6‒03 monitor, Treaton Electronics, Russia), the expired tidal volume (VTe) using an expiratory flow sensor of the ventilator through a mouthpiece (Savina Select E, Drager, Germany), and the EEP using a pressure transducer (AICU Vision, Bark Technology, Kazakhstan). Data on oximetry, capnography, Ti, and Te were analysed using dedicated software (SideStream Capno, Treaton Electronics, Russia). All measurements were recorded within the stable respiratory pattern viewed on capnography. The catheter was flushed with air on demand. Additionally, we recorded patient comfort using a visual analogue scale (from 1 to 10, where 10 meant full comfort) and asked the subjects to describe their sensations throughout the study period.

After the initial measurements, we established a high-flow oxygen device through a nasal cannula (Hifent HUMID-BH, RespirCare Medical Solutions, China) at a flow rate of 30 L/min and a preset oxygen fraction (F_D_O_2_) of 40%. Cannula sizes were selected according to the manufacturer’s manual (5 mm diameter for all subjects). We performed 12 series of measurements with preset flow rates (PFR) of 30, 60, and 80 L/min combined with preset F_D_O_2_ values of 40, 60, 80, and 100% at each flow rate. In each series, we asked the subjects to breathe in four different respiratory patterns: quiet breathing with a closed mouth (CM), quiet breathing with an open mouth (OM), hyperpnea with a closed mouth (HCM), and hyperpnea with an open mouth (HOM). For the hyperpnea series, the subjects were asked to breathe approximately two times deeper (subjects were trained with a volumetric assessment) at a rate of 30 breaths per minute, and the RR in these series was controlled with a multigas monitor. For the open mouth series (OM and HOM), the volunteers were asked to breathe through the mouth. We performed all measurements the same as in the baseline period at each series combined with each respiratory pattern (48 steps total), except VTe during OM and HOM (unable to perform the correct VTe measurements). All the measurements were recorded after 2 min of the current step. The wash-out period between each step was approximately 2 min. The total duration of the measurements for a subject was approximately 4 h. We also assessed oxygram and capnogram waveforms during all steps of the study. Additionally, after completing all the measurements, the volunteers were asked to describe their feelings in their own words. We calculated the modified ventilatory ratio (mVR) at the end of the CM and HCM steps with the following equation: mVR = [RR * VTe * (PetCO_2_ (mmHg)—4 mmHg)]/[predicted body weight (kg) * 100 (ml/kg/min) * 37.5 mmHg]. We calculated the mean volunteer flow by dividing VTe by Ti at the end of each step for CM and HCM with the assumption that VTe was close to the inspired tidal volume. The mean subject flow was compared with the preset flow rate and VAS comfort score at all steps.

### Statistical analysis

Given the physiological design of the study, we did not perform a formal sample size calculation. We used a sample size of 10 patients for this study, in accordance with previous investigations on this topic [11]. Descriptive statistics included proportions for categorical variables and medians with interquartile ranges (IQRs) for continuous variables. No imputation was performed for missing data. Comparisons between steps were performed using the Friedman test for continuous variables and the Cochran’s Q test for binary values. Post-hoc pairwise comparisons were made using the Friedman test with Bonferroni correction for multiple comparisons (to reduce type I errors). A two-sided *p* < 0.05 was considered statistically significant. All the statistical analyses were performed using SPSS 26.0 (IBM, Armonk, NY, USA).

## Results

We included 20 healthy volunteers (aged 24 (22–36) years, 11 men, height of 176 (167–181) cm, weight of 76.5 (66.0–85.0) kg, body mass index of 25.6 (21.2–28.5) kg/m^2^, two mild smokers). One volunteer refused to continue the study at a PFR of 80 L/min because of severe discomfort but completed all steps with PFR of 30 and 60 L/min (36 steps).

### Preset F_D_O_2_ of 100%

#### Influences of the PFR and breathing pattern on FiO_2_

**Fig. 1:**
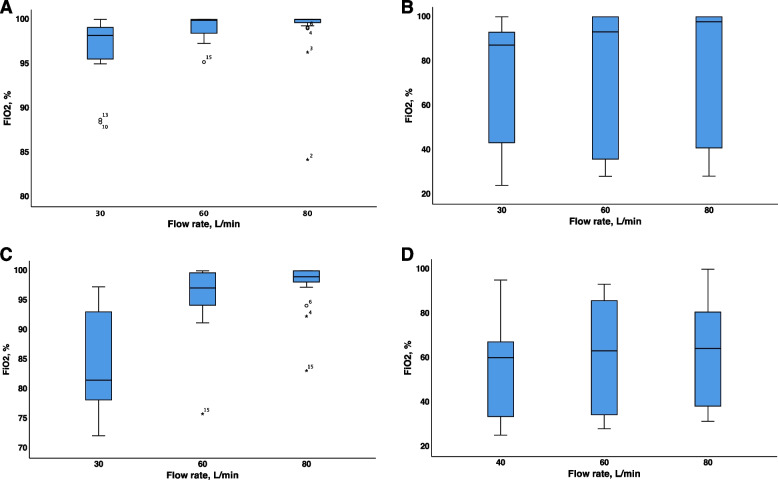
Measured inspiratory oxygen fraction in the hypopharynx at various breathing patterns of volunteers with different preset flow rates and preset inspiratory oxygen fraction of 100%. **A** CM—quiet breathing with closed mouth. **B** OM—quiet breathing through open mouth. **C** HCM—hyperpnea with closed mouth. **D** HOM—hyperpnea through open mouth. Abbreviations: FiO_2_—measured inspiratory oxygen fraction

An increase in the PFR from 30 to 80 L/min during all phases led to a significant increase in FiO_2_ (*p* < 0.05 for all comparisons). During the CM phase, PFR of 60 and 80 L/min provided the preset F_D_O_2_; at the PFR of 30 L/min, the FiO_2_ was close to 100%. An increase in the PFR from 30 to 80 L/min in the OM phase increased the median FiO_2_ to 97.8%, but with a wide interquartile range (IQR) (39.4–100.0%), an increase in the PFR from 30 to 60 L/min in the OM phase had a similar effect. During the HCM phase, an increase in the PFR from 30 to 60 and to 80 L/min provided an FiO_2_ close to 100%. During the HOM pattern, only an increase from the PFR from 30 to 80 L/min led to a significant increase in FiO_2_, but it had a wide range of FiO_2_ values and never reached the preset level of F_D_O_2_. OM and HOM patterns provided less FiO_2_ than CM and HCM patterns, respectively, with very wide IQRs (Fig. 1, Table 1).

**Table 1:** Gas composition, pressure in the hypopharynx, and respiratory parameters at different preset flows, 100% inspiratory fraction of oxygen, and various breathing patterns of volunteers

Variable	Breathing Pattern (BP)	HFNC flow rate			P between preset flow
		**30 L/min**	**60 L/min**	**80 L/min**	
FiO_2_, %	**CM**	98.3 (95.3–99.6)	99.9 (98.2–100.0)*	100.0 (99.3–100.0)**	**< 0.001**
	**OM**	84.8 (41.7–94.0)†	91.2 (35.6–100.0)*†	97.8 (39.4–100.0)**†	**0.021**
	**HCM**	81.4 (77.2–94.3) ‡	96.9 (94.1–99.8)*	99.0 (98.1–100.0)**	**< 0.001**
	**HOM**	59.2 (32.8–67.2)#	66.6 (34.1–85.9)#	64.3 (36.3–82.3)**#	**0.014**
	**p between all BP**	**< 0.001**	**< 0.001**	**< 0.001**	
Abnormal oxygram waveform, n (%)	**CM**	0	0	0	NA
	**OM**	7 (35)†	11 (55)†	11 (55)†	0.069
	**HCM**	13 (65) ‡	15 (75) ‡	16 (80) ‡	0.247
	**HOM**	15 (75)	17 (85)	17 (85)	0.135
	**p between all BP**	** < 0.001**	**< 0.001**	**< 0.001**	
FeCO_2_, %	**CM**	2.9 (2.6–3.6)	2.6 (2.1–3.4)*	2.1 (1.8–3.1)**	**< 0.001**
	**OM**	2.6 (2.2–2.9)†	2.2 (1.6–2.9)*†	2.0 (1.7–2.4)**	**0.001**
	**HCM**	2.4 (2.2–3.2) ‡	2.0 (1.6–2.6)*‡	1.7 (1.4–2.5) **‡	**< 0.001**
	**HOM**	1.9 (1.7–2.2)#	1.8 (1.5–2.3)*	1.4 (1.1–1.7)**§#	**< 0.001**
	**p between all BP**	** < 0.001**	** < 0.001**	** < 0.001**	
Expiratory pressure, cmH_2_O	**CM**	3.8 (3.1–6.6)	7.6 (6.4–11.4)*	11.1 (7.7–14.8)** §	**< 0.001**
	**OM**	1.2 (0.7–1.4)†	1.5 (0.9–2.3)†	1.5 (0.8–2.9)†	0.102
	**HCM**	5.8 (3.5–8.2) ‡	9.4 (6.8–13.1)* ‡	12.7 (8.2–18.1)** § ‡	**< 0.001**
	**HOM**	1.5 (1.1–1.7)#	2.4 (1.4–2.7)*#	3.0 (1.3–3.8)**#	**0.005**
	**p between all BP**	** < 0.001**	** < 0.001**	** < 0.001**	
Delta pressure, cmH_2_O	**CM**	3.5 (2.5–5.8)	3.5 (1.9–9.4)	4.5 (1.4–11.4)	0.500
	**OM**	1.1 (0.8–1.5)†	1.2 (0.7–1.7)†	1.5 (0.6–3.7)†	0.584
	**HCM**	7.1 (4.5–10.0) ‡	9.9 (6.1–14.7) ‡	12.2 (9.1–18.6) **‡	**0.009**
	**HOM**	1.8 (1.3–3.2)#	2.1 (1.6–3.7)#	1.9 (1.1–4.4)#	0.383
	**p between all BP**	** < 0.001**	** < 0.001**	**< 0.001**	
Minimal inspiratory pressure, cmH_2_O	**CM**	0.5 (0.3–1.5)	3.9 (2.3–5.1)*	5.4 (1.5–8.0)**	**0.001**
	**OM**	0.3 (0.0–0.4)	0.3 (0.0–0.9)†	0.2 (−0.2–1.0)†	0.212
	**HCM**	−1.9 (−3.9–0.3) ‡	0.6 (−2.5–2.5)*‡	0.5 (−2.4–2.7)**‡	**0.025**
	**HOM**	−0.5 (−1.8–0.3)	−0.1 (−1.3–0.5)	0.0 (−0.7–0.5)	0.097
	**p between all BP**	** < 0.001**	** < 0.001**	**< 0.001**	
Exhaled tidal volume, mL	**CM**	711.0 (608.3–790.0)	948.0 (715.0–1204.8)*	948.0 (869.0–1422.0)**	**< 0.001**
	**HCM**	790.0 (681.4–898.6)	987.5 (869.0–1194.9)*	1027.0 (869.0–1264.0)**	**< 0.001**
	**p between BP**	0.059	0.808	0.819	
Respiratory rate, min^−1^	**CM**	11.0 (9.3–16.0)	10.5 (8.0–14.0)	10.0 (7.0–15.0)**	**0.043**
	**OM**	14.0 (11.0–16.8)	15.0 (12.0–18.9)	16.0 (12.0–19.0)	0.531
	**HCM**	27.0 (24.3–30.0) ‡	30.0 (28.0–32.8)* ‡	30.0 (28.0–33.0)** ‡	**0.004**
	**HOM**	30.0 (28.0–31.0) #	31.0 (30.0–33.0)*	32.0 (29.0–35.0)**	**0.006**
	**p between BP**	** < 0.001**	** < 0.001**	** < 0.001**	
Ventilatory ratio (modified), AU	**CM**	0.62 (0.49–0.91)	0.59 (0.38–0.88)	0.50 (0.40–0.73)	0.076
	**HCM**	1.13 (0.54–1.54) ‡	1.22 (0.96–1.50) ‡	1.05 (0.71–1.84) ‡	0.949
	**p between BP**	** < 0.001**	** < 0.001**	** < 0.001**	
Expiratory time, s	**CM**	3.4 (2.2–5.4)	3.4 (2.9–6.0)	3.7 (2.5–5.8)	0.759
	**HCM**	1.2 (1.0–1.3) ‡	1.0 (0.9–1.2) ‡	1.0 (0.9–1.2) ‡	0.063
	**p between all BP**	** < 0.001**	** < 0.001**	** < 0.001**	
Inspiratory time, s	**CM**	1.7 (1.2–2.0)	1.7 (1.1–2.0)	1.8 (1.2–1.9)	0.570
	**HCM**	1.0 (1.0–1.2) ‡	0.9 (0.8–1.0) *‡	0.9 (0.8–1.0)**‡	**0.002**
	**p between all BP**	** < 0.001**	** < 0.001**	** < 0.001**	
Ti/Te, ratio	**CM**	0.47 (0.30–0.65)	0.41 (0.34–0.50)	0.49 (0.29–0.60)	0.311
	**HCM**	0.90 (0.77–1.00) ‡	0.91 (0.83–1.00) ‡	0.83 (0.76–1.00) ‡	0.156
	**p between all BP**	**0.001**	** < 0.001**	**0.001**	
Visual-analog scale of comfort, points	**CM**	8.0 (7.3–9.8)	7.0 (5.3–8.8)*	6.0 (4.0–8.0)** §	**< 0.001**
	**OM**	8.0 (7.0–9.8)	6.5 (5.3–8.8)*	6.0 (3.0–8.0)** §	**< 0.001**
	**HCM**	8.0 (6.3–9.0)	6.5 (5.0–8.0)*	6.0 (3.0–8.0)**	**< 0.001**
	**HOM**	8.0 (6.3–9.0)	6.5 (5.0–8.0)*	5.5 (3.0–8.0)**	**< 0.001**
	**p between all BP**	**0.030**	0.051	0.866	
Mean inspiratory flow, L/min	**CM**	26.3 (20.3–33.6)	39.6 (27.1–49.8)*	44.8 (30.1–51.8)**	**0.003**
	**HCM**	43.6 (40.3–52.1) ‡	61.6 (50.0–77.0)* ‡	68.4 (56.9–90.9)** ‡	**< 0.001**
	**p between all BP**	** < 0.001**	**0.001**	**0.001**	
Preset flow/mean inspiratory flow	**CM**	1.14 (0.89–1.48)	1.51 (1.20–2.21)	1.79 (1.54–2.66)** §	**0.001**
	**HCM**	0.69 (0.58–0.74) ‡	0.97 (0.78–1.20)* ‡	1.17 (0.88–1.41)** ‡	**< 0.001**
	**p between all BP**	** < 0.001**	**0.001**	**0.001**	

Data are presented as median (interquartile range)

*HFNC* high flow oxygen through nasal cannula, *BP* breathing pattern, *CM* quiet breathing with closed mouth, *OM* quiet breathing with opened mouth, *HCM* hyperpnea with closed mouth, *HOM* hyperpnea with opened mouth, FiO_2_ measured inspiratory oxygen fraction, FeO_2_ measured expiratory oxygen fraction, FeCO_2_ end-expiratory carbon dioxide fraction, *AU* arbitrary units, *VAS* visual-analog scale (10 points—maximal comfort, 1 point—maximal discomfort), *Te* expiratory time, *Ti* inspiratory time

Significant differences (Friedman test with Bonferroni correction for multiple comparisons or Cochran’s Q test, where appropriate with *p* < 0.05) between preset flows with stable respiratory pattern: * 30 vs 60 l/min, ** 30 vs 80 l/min, § 60 vs 80 l/min. Significant differences (Friedman test with Bonferroni correction for multiple comparisons or Cochran’s Q test, where appropriate with *p* < 0.05) between different respiratory patterns with a constant preset flow: †CM vs OM, ‡CM vs HCM, #HCM vs HOM

Significant differences in p values are highlighted in bold

**Fig. 2:**
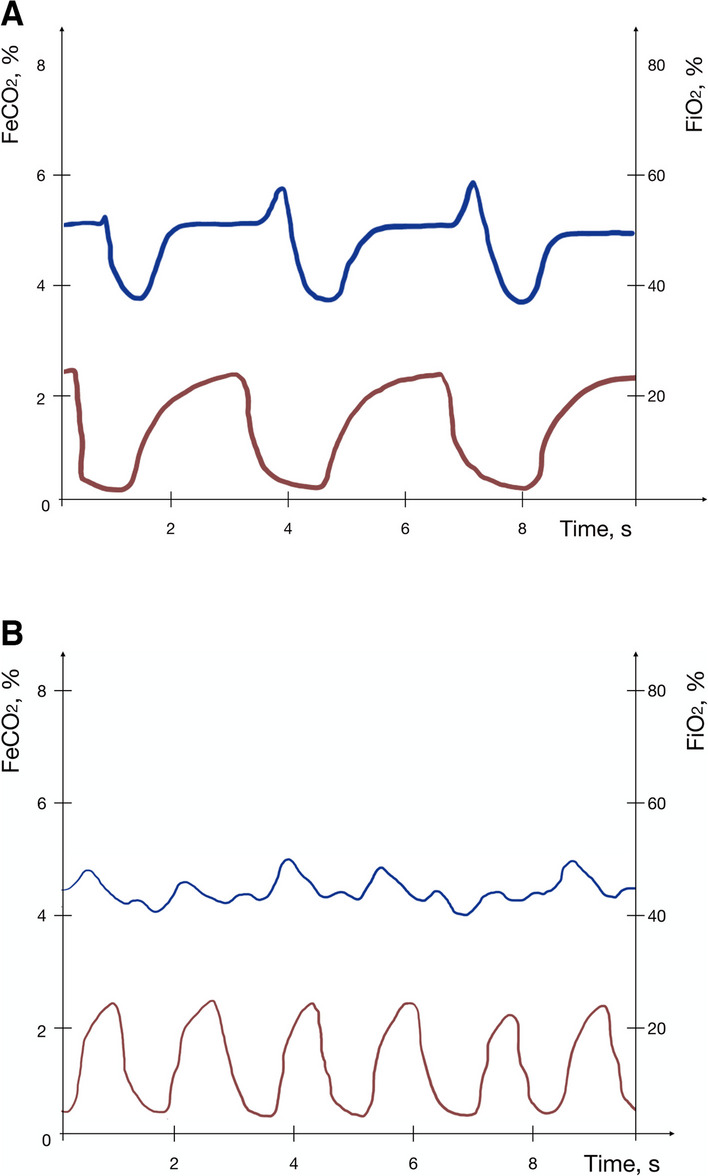
Capnogram and inverted oxygram during hyperpnea through open mouth. **A** inverted oxygram pattern. **B** combined oxygram pattern. Blue line: oxygram, Red line: capnogram

We discovered a variety of “abnormal” oxygram waveforms during the OM, HCM, and HOM patterns: inverted (almost copying the capnogram shape), flat (without prominent inspiratory and expiratory phases), and combined (combination of inverted and flat) (Fig. 2). The prevalence of abnormal oxygrams was zero during CM breathing and very common during HCM, HOM, and OM breathing (less common than during HCM and HOM breathing) at all PFR (Table 1). An increase in the PFR did not affect the oxygram waveform (Table 1).

### Influences of the PFR and breathing pattern on FeCO_2_

**Fig. 3:**
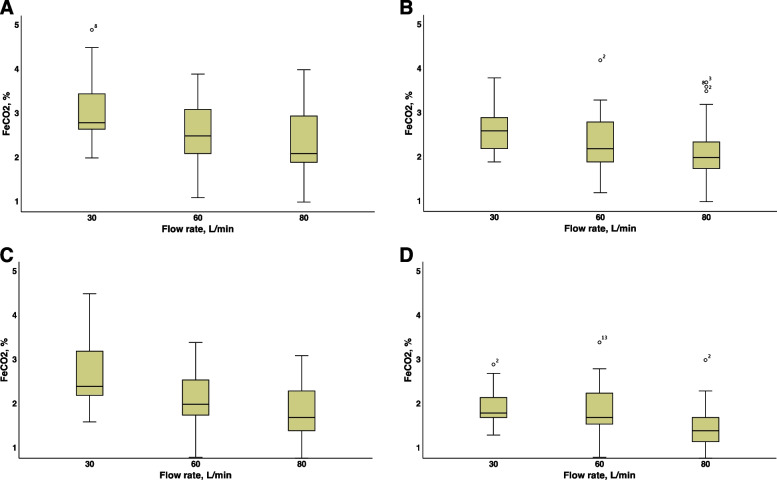
Measured end-expiratory carbon dioxide fraction in the hypopharynx at various breathing patterns of volunteers with different preset flow rates and preset inspiratory oxygen fraction of 100%. **A** CM—quiet breathing with closed mouth. **B** OM—quiet breathing through open mouth. **C** HCM—hyperpnea with closed mouth. **D** HOM—hyperpnea through open mouth. Abbreviations: F_E_CO_2_—end-expiratory carbon dioxide fraction

The initial FeCO_2_ was 4.7 (4.3–5.3)%. The initiation of HFNC therapy resulted in a significant decrease in FeCO_2_ (*p* < 0.001). An increase in the PFR from 30 to 80 L/min resulted in a significant decrease in FeCO_2_ during all breathing patterns (*p* < 0.01 between all comparisons). Compared with the CM pattern, the OM pattern significantly decreased the FeCO_2_ level; compared with the CM pattern, the HCM pattern decreased the FeCO_2_ level; and compared with the HCM pattern, the HOM pattern decreased the FeCO_2_ level (Fig. 3, Table 1).

### Influences of the PFR and breathing pattern on the end-expiratory pressure and delta pressure

**Fig. 4:**
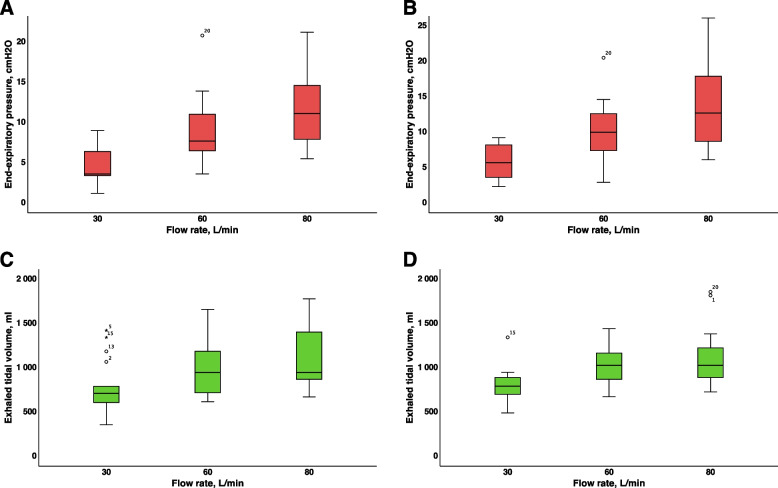
Measured end-expiratory pressure in the hypopharynx (**A**, **B**) and measured expiratory tidal volume (**C**, **D**) at various breathing patterns of volunteers and different preset flow rates and preset inspiratory oxygen fraction of 100%. **A**, **C** during CM—quiet breathing with closed mouth. **B**, **D** during HCM -hyperpnea with closed mouth. Abbreviations: EEP—end-expiratory pressure, VTe—expiratory tidal volume

An increase in the PFR to 30, 60 and 80 L/min during the CM pattern resulted in a significant increase in the EEP: 3.8 (3.1–6.6) mbar), 7.6 (6.4–11.4) mbar, and 11.1 (7.7–14.8) mbar, respectively (*p* < 0.001 for all comparisons). A change in the breathing mode to OM significantly decreased the EEP, and an increase in the PFR did not influence the EEP during OM (Fig. 4A and B, Table 1). A change in the breathing mode from CM to HCM resulted in an increase in the EEP in the hypopharynx (*p* < 0.05 for all comparisons) (Fig. 4A and B, Table 1). An increase in the PFR during the HCM pattern significantly increased the EEP (*p* < 0.001 for all comparisons) (Fig. 4A and B, Table 1). The EEP during the HOM pattern was significantly lower than that during the HCM pattern (*p* < 0.001 for all comparisons). An increase in the PFR significantly increased the EEP during the HOM pattern (*p* = 0.005 for all comparisons) (Table 1).

DeltaP was lower during OM than during CM breathing, higher during HCM than during CM breathing, and lower during HOM than during HCM breathing (Table 1). DeltaP significantly increased with the increasing PFR only during the HCM phase (Table 1).

### Influences of the PFR and breathing pattern on the exhaled tidal volume

The initial VTe before the study was 422.7 (347.6–509.6) mL. VTe during CM with an F_D_O_2_ of 100% increased from the baseline value to 711.0 (608.3–790.0), 948.0 (715.0–1204.8), and 948.0 (869.0–1422.0) mL at PFR of 30, 60, and 80 L/min, respectively (*p* < 0.001) (Fig. 4C and D, Table 1). An increase in the PFR during the HCM pattern also increased VTe (*p* < 0.001). The VTe during the HCM and CM patterns did not differ at any of the PFR (*p* > 0.05) (Fig. 4C and D, Table 1).

### Influences of the PFR and breathing pattern on the expiratory and inspiratory times

**Fig. 5:**
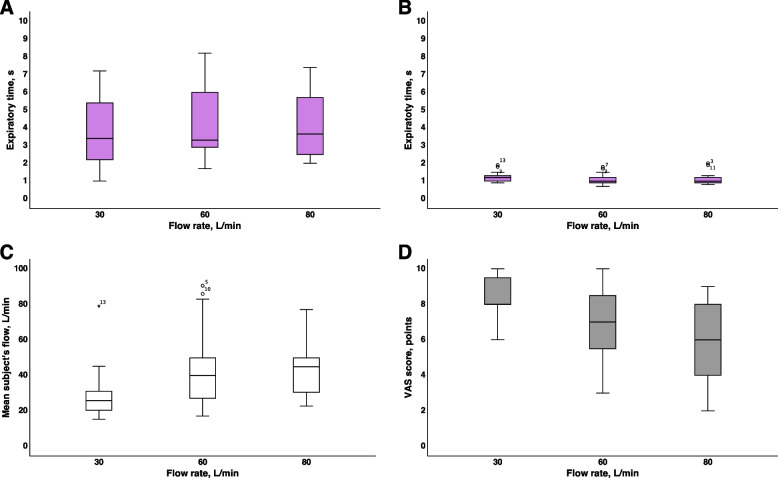
**A**, **B** Measured expiratory time at various breathing patterns of volunteers and different preset flow rates and preset inspiratory oxygen fraction of 100%. **A** CM—quiet breathing with closed mouth. **B** HCM—hyperpnea with closed mouth. C Mean subject’s expiratory flow during quiet breathing with closed mouth at different preset flow rates and preset inspiratory oxygen fraction 100%. **D** Comfort expressed as visual-analog scale during quiet breathing with closed mouth at different preset flow rates and preset inspiratory oxygen fraction 100%. Abbreviations: VAS—visual-analog scale (10 points—maximal comfort, 1 point—maximal discomfort)

The initial Te before the study was 2.2 (2.0–2.7 s). The Te during the CM pattern at PFR of 30 L/min increased to 3.4 (2.2–5.4) s (*p* = 0.005) (Fig. 5A, Table 1). An increase in the PFR to 60 and 80 L/min did not change the Te further during the CM phase (Fig. 5A, Table 1). Compared with the CM mode, switching the breathing mode to HCM resulted in a significant decrease in the Te at all PFR (Fig. 5B, Table 1). An increase in the PFR did not change the Te further during the HCM phase (Fig. 5B, Table 1). The initial Ti before the study was 1.7 (1.1–1.9 s). Ti during the CM pattern and an F_D_O_2_ of 100% did not change at any of the flow rates (Table 1). A change in the breathing mode from CM to HCM resulted in a significant decrease in Ti at all PFR (Table 1). An increase in the PFR from 30 to 60 and 80 L/min during the HCM pattern shortened the Ti (*p* = 0.002) (Table 1).

### Influences of the PFR and breathing pattern on the respiratory rate

The initial RR was 14 (12–16) min^−1^. The RR during the CM pattern at a flow rate of 30 L/min decreased to 11.0 (9.3–14.0) min^−1^ (*p* = 0.012). An increase in the PFR from 30 to 80 L/min decreased the RR further to 10.0 (7.0–15.0) (*p* = 0.043) (Table 1). A change in the breathing mode from CM to OM resulted in an increase in the RR at PFR of 60 and 80 L/min, which did not reach statistical significance (*p* = 0.058 and *p* = 0.059, respectively) (Table 1). The RR was significantly increased during the HOM breathing pattern compared with that during the HCM breathing pattern at a flow rate of 30 L/min (Table 1).

### Influences of the PFR and breathing pattern on the modified ventilatory ratio

The initial mVR was 0.71 (0.58–0.85). The mVR during the CM pattern at all flow rates did not change compared with the initial mVR (Table 1). Switching the breathing pattern from CM to HCM was associated with an increase in the mVR at all preset flow rates (Table 1).

### Influences of the PFR and breathing pattern on the subject’s comfort

The VAS comfort score during the CM breathing pattern at a flow rate of 30 L/min was 8.0 (7.3–9.8) points. An increase in the PFR to 60 and 80 L/min during the CM breathing pattern resulted in a significant decrease in the subject’s comfort to 7.0 (5.3–8.8) and 6.0 (4.0–8.0) points, respectively (*p* < 0.001) (Fig. 5C, Table 1). The same dynamics of the subject’s comfort were observed for all other patterns (Table 1). A change in the breathing pattern from CM to HCM and from OM to HOM did not change the subject’s comfort (Table 1).

The mean subject expiratory flow (EFmean) increased as the PFR increased during the CM and HCM patterns (Table 1). The HCM pattern was associated with a higher EFmean than CM at all PFR (*p* = 0.001) (Table 1).

The VAS comfort score corresponds to the ratio of the PFR to the EFmean of a volunteer during CM: the maximum comfort of the subject was observed at a ratio close to 1:1, and the maximum discomfort was observed at a ratio of approximately 2:1 (Fig. 5D, Table 1).

The subjects’ answers concerning their discomfort are presented in the Supplemental material (Table S5).

### Physiological variables at preset F_D_O_2_ values of 40%, 60%, and 80%

The dynamics and significant differences of the measured values with the preset F_D_O_2_ of 40, 60 and 80% were similar to those of the PFR increase and changes in the breathing patterns with the preset F_D_O_2_ of 100% The FiO_2_/F_D_O_2_ ratio decreased with increasing F_D_O_2_. For example, an increase in F_D_O_2_ from 40 to 100% during HOM at PFR of 80 and 60 L/min resulted in a decrease in the median FiO_2_/F_D_O_2_ ratio from 85 to 64% (Tables 1–2, S1–S3; Figs. S1–S2). An increase in the PFR did not influence the measured RR with the preset F_D_O_2_ of 40, 60 and 80% during all breathing patterns; RR did not differ between the CM and OM or between the HCM and HOM patterns. All data on the preset F_D_O_2_ values of 40, 60, and 80% are presented in Supplemental Tables S1, S2, and S3, respectively.

### Influences of the preset F_D_O_2_ at constant PFR on FeCO_2_, the mVR, and the comfort of the subject

The increase in F_D_O_2_ during the CM pattern at constant PFR decreased the FeCO_2_ and mVR in all comparisons (Supplemental Table S4 and Figures S3–S5). The FeCO_2_ significantly decreased with the increase in the preset F_D_O_2_ also during OM, HCM, and HOM patterns at constant PFR (*p* < 0.05 for all comparisons). Changes in the F_D_O_2_ significantly affected the mVR also during HCM pattern (*p* < 0.05). The VAS score decreased significantly with increasing F_D_O_2_ only at the PFR of 80 L/min during the CM (Table S4, Fig. S3C), OM (*p* < 0.001), and HOM (*p* = 0.004) patterns. All other variables during stable PFR were independent of F_D_O_2_ (*p* > 0.05 for all comparisons).

## Discussion

Most of the studies on respiratory physiology during HFNC therapy in healthy volunteers and patients have focused on the EEP, breathing pattern changes, and work of breathing. The heterogeneous data for these parameters could be explained by heterogeneous sites of measurements: the placement of the tip of the probe in different parts of the volunteer’s upper airways (nasopharynx, nasal cavity, oropharynx, or hypopharynx) or inside the mannequin. We first focused on the gas composition in the hypopharynx and the influences of the combination of the PFR, breathing pattern, and F_D_O_2_ on FiO_2_ (including oxygram shape), FeCO_2_, EEP, and VTe.

### FiO_2_

Data on FiO_2_ measurements inside airways close to the trachea or inside the trachea in healthy subjects and patients with ARF are limited. Similar FiO_2_ values ​​were obtained in healthy volunteers, models, or patients, when the catheter was placed in the hypopharynx or inside the trachea (5–6), but the FiO_2_ values measured in the nasopharynx was lower [7].

We conducted the first study in which the resulting FiO_2_ in the hypopharynx at different respiratory patterns, PFR, and F_D_O_2_ values were evaluated. We found a very high range of FiO_2_ within the combination of F_D_O_2_ at different PFR and breathing patterns. These results may call into question the accuracy of the measurement of the ROX index and SpO_2_/FiO_2_ during HFNC therapy in clinical studies, especially with respect to the OM and HOM patterns [16]. Sun YH et al. examined a lung model and observed a relationship between the PFR, F_D_O2, and peak inspiratory flow, in which resulting FiO_2_ was much lower than that in our data [17].

FiO_2_ measurements in the hypopharynx during HFNC therapy with the OM, especially the HOM breathing pattern, are challenging due to “abnormal” oxygram waveforms during inspiration. The inverted oxygram may be a consequence of the Venturi effect with air entrainment through an open mouth and collision of the expiratory and inspiratory flows. Future studies are needed to assess the clinical relevance of these findings.

### FeCO_2_

*Bench studies* have shown an improvement in CO_2_ clearance during HFNC therapy in the nasal cavity, pharynx, and trachea (8–10), which is more prominent with higher PFR and prolonged Te (decreased RR) [9]. Onodera Y et al. reported that CO_2_ clearance during the OM pattern was higher than during the CM pattern, which is consistent with our data [10].

Although bench studies revealed a flow-dependent improvement in CO_2_ clearance, the conclusions from *volunteer studies* were not as clear. Moller W et al. confirmed their previous bench data [8] on flow-dependent CO_2_ clearance and clearance half-times using scintigraphy [18]. In addition, other studies did not identify changes in arterial, tissue, and capillary CO_2_ levels (PaCO_2_, PtCO_2_, or PcCO_2_, respectively), or FeCO_2_ during HFNC [7, 19, 20]. If constant PaCO_2_/PtCO_2_/PcCO_2_ levels could be explained by a combination of a reduced VD and a decrease in the RR during HFNC therapy and, as a consequence, stable VA, then a constant PaCO_2_/PtCO_2_/PcCO_2_ during unchanged MV with increased VD washout and an increased VT could be explained only by an incorrect VT measurement or probable increased alveolar VD due to the CPAP-like effect with alveolar overdistension [20]. In our study, we measured FeCO_2_, which is close to PaCO_2_, and observed a decrease in FeCO_2_ during constant CO_2_ production, suggesting increased VA. The significant decrease in the RR with decreasing FeCO_2_ in our study could be explained by a significant increase in the VT during HFNC therapy. We observed a pronounced effect on CO_2_ washout, which was influenced by FiO_2_ levels and the respiratory pattern of the subject (CM, OM, HCM, or HOM). To our knowledge, our study is the first to compare FeCO_2_ during four different breathing patterns.

Numerous *clinical studies* of hypercapnic patients have confirmed data on improved CO_2_ washout in patients, revealing a decrease in PaCO_2_/PtCO_2_/PcCO_2_ [21–30]. However, some studies on AHRF did not find a decrease in PaCO_2_/PtCO_2_/PcCO_2_, which could be explained by constant VA in these patients or increased CO_2_ production [31, 32].

The following question remains unanswered: does end-tidal CO_2_ during HFNC therapy reflect only alveolar gas or mixed gas from alveoli and entrained gas from high-flow cannulas?

### VT

High tidal volumes during HFNC may injure the lungs through lung overinflation (strain) or patient self-inflicted lung injury (P-SILI) [33, 34]. VT above 9.5 mL/kg were found to be a predictor of NIV failure in a single multicentre observational study of patients with moderate-to-severe hypoxemic ARF treated with NIV [35].

The VT measurement methods used during HFNC therapy in published studies are not ideal. These factors may lead to conflicting results showing the opposite direction of VT changes with an increase in the inspiratory flow rate. We subdivided the discussion of VT changes during HFNC therapy by the VT measurement method.

Data on VT measurements during HFNC therapy obtained using electrical impedance tomography [31, 32, 36–39] and inductive plethysmography [19, 21, 40] have shown various results in healthy volunteers and patients with ARF. Studies using thoracic bioimpedance (ExSpiron 2Xi, Senzime, Sweden) in healthy volunteers [20] and volumetric measurements during HFNC interruption in patients with ARF [41] showed an increase in the VT of up to 11 ml/kg IBW, which is consistent with our data.

To the best of our knowledge, a time-of-flight camera is the most accurate noninvasive method for VT measurements in nonintubated patients. A single study used this method to assess VT during noninvasive respiratory support [42]. This study revealed that an increase in the PFR and/or an increase in the subject’s respiratory drive was associated with an increase in VT to 913 (680–1166) mL. Moreover, with a PFR of 30 L/min, 50% of the subjects had a VT greater than 10 mL/kg, and 15% had a VT > 15 mL/kg; however, with a PFR of 60 L/min, 65% of the subjects had a VT greater than 10 mL/kg, 35% had a VT greater than 15 mL/kg, and 10% had a VT greater than 20 mL/kg. These results are consistent with our data.

### End-expiratory pressure and end-expiratory volume

Bench studies have shown highly heterogeneous values for EEP during the CM phase, ranging from 0–3 cm H_2_O [38] when measured inside a mannequin “nasopharynx” to 16 cm H_2_O at a PFR of 60 L/min when measured inside a lung model [10, 17]. Substituting appropriate numbers from our study during the CM pattern to their equation, we obtain an EEP level equal to 14.5 cmH_2_O inside the lung model, which corresponds to our data. In our study, we observed a long phase of positive EEP, which contradicts the findings of bench studies that documented only a short (“peak”) EEP [17].

Volunteer studies also revealed heterogeneous data for EEP values. Most studies have measured the EEP in the nasopharynx or distal part of the nasal cavity. These studies reported concordant results: EEP levels were approximately the PFR divided by ten [38, 43–45]. In contrast, two studies that measured EEP in the oropharynx [46] or the hypopharynx [7] reported higher EEP values during the CM pattern, which were close to our data but presented high variability [7]. PEEP levels also depend on the cannula size: the EEP level was higher with a 5 mm cannula than with a 4 mm or 3 mm cannula [47]. Moreover, the volunteers in the Chinese study [48] had higher PEEP levels than those in the European study did, possibly due to the anatomical features of the upper airways.

Clinical studies using EIT revealed an increase in end-expiratory lung impedance (EELI) in all studies [22, 32, 33, 37, 39, 48], with greater EELI at higher PFR. Surprisingly, in two clinical studies, the EEP was lower than that in volunteer studies (including our study). This difference can be explained by partial mouth opening during HFNC therapy in these patients [6, 37]. Villalba DS have found no differences between tracheal and pharyngeal pressures during HFNC with different PFR during the CM and OM patterns in patients with a tracheostomy during the process of decannulation [12]. The clinical data on EEP are discussed in detail in a dedicated review [49].

### Subject’s comfort and work of breathing (WOB)

In the previous studies, the WOB in healthy subjects was not significantly different at the range of PFR of 10–40 L/min but increased significantly at higher PFR or when larger cannulas were used [19, 38, 47]. In healthy volunteers, the WOB was higher during HFNC therapy at 40 and 60 L/min during the CM pattern than during CPAP therapy at 4 cmH_2_O for a single breath, also the WOB during the CM pattern was greater than that during the OM pattern [38]. In contrast, we found that about half of the volunteers preferred the CM pattern as more comfortable. In AHRF patients, dyspnea (Borg score) was less pronounced during HFNC therapy than during low-flow oxygen therapy [37] but was more pronounced if excessive PFR were used, and it was significantly higher if the PFR was 1.5 L/min per ideal body weight [48], which is comparable to our VAS data obtained at a PFR of 80 L/min in healthy volunteers.

Our study has several strengths: 1. simultaneous monitoring of capnogram, oxygram, pressure, and volume measurements and VAS comfort during HFNC therapy; 2. comparisons of three different PFR, including an unusually high flow rate of 80 L/min; 3. The use of combinations of different F_D_O_2_ values and PFR to reveal the influences of these combinations on the gas composition in the hypopharynx; 4. measurements were made in the hypopharynx, close to vocal cords, which made it more reliable for clinical practice; 5. The use of different breathing patterns, including not only CM and OM but also hyperpnea during CM and OM; and 6. The calculation of the mVR at all preset combinations of flow rates, F_D_O_2_ values, and breathing patterns.

The limitations of the study include the following: 1. The VT measurement technique may introduce some bias due to the Venturi effect with gas entrainment and changes in the breathing pattern during the VT measurement; we mitigated this bias by training volunteers in the optimal measuring technique and excluded subjects with abnormal oxygrams in whom the Venturi effect was evident from the analysis. 2. The results may not be fully translated into clinical practice for patients with ARF due to pathophysiological changes.

## Conclusions

HCM, OM, and HOM breathing patterns decreased FiO_2_ and F_E_CO_2_ in the hypopharynx of healthy subjects (which was more pronounced during the OM and HOM patterns). The FiO_2_ level in the hypopharynx during the OM and HOM patterns is unpredictable at all PFR. A PFR of 60 L/min was sufficient to maintain FiO_2_ close to F_D_O_2_ during the CM and HCM patterns. HFNC within the CM and HCM provided flow-dependent CPAP-effects over a wide range and could be associated with lung hyperinflation. An excessive PFR led to discomfort.

## Supplementary Information

Supplementary Material 1.

## Data Availability

The datasets used and/or analysed during the current study are available from the corresponding author upon reasonable request.

## Abbreviations

AHRF: Acute hypoxemic respiratory failure
CM: Closed mouth,
COPD: Chronic obstructive pulmonary disease
CO_2_: Carbon dioxide
CPAP: Constant airway pressure
DeltaP: Delta of pressure
EELI: End-expiratory lung impedance
EEP: Measured end-expiratory pressure in the hypopharynx
Efmean: Mean expiratory flow
F_D_O_2_: Preset oxygen fraction on HFNC device
F_E_CO_2_: Measured expiratory carbon dioxide fraction in the hypopharynx
FiO_2_: Measured inspiratory oxygen fraction in the hypopharynx
HCM: Combination of CM with hyperpnea
HFNC: High flow through nasal cannula
HOM: Combination of OM with hyperpnea
IBW: Ideal body weight
IQR: Interquartile range
MV: Minute ventilation
OM: Open mouth
O_2_: Oxygen
PaCO_2_: Arterial carbon dioxide partial pressure
PcCO_2_: Capillary carbon dioxide partial pressure
PEEP: Positive end-expiratory pressure
PetCO_2_: End-expiratory carbon dioxide partial pressure
P-SILI: Patient self-inflicted lung injury
PtCO_2_: Tissue carbon dioxide partial pressure
ROX index: Calculated as SpO_2_/FiO_2_/RR
RR: Respiratory rate
SpO_2_: Peripheral oxygen saturation
Te: Expiratory time
Ti: Inspiratory time
VAS: Visual-analog scale
V’CO_2_: CO_2_ production,
VD: Dead space volume
VR: Ventilatory ratio
VT: Tidal volume
Vte: Exhaled measured tidal volume
WOB: Work of breathing
