# Supplementary material for: Gas composition and pressure in the hypopharynx during high-flow oxygen therapy through a nasal cannula in healthy volunteers with different breathing patterns
Source: BMC Anesthesiol. 2025 Aug 23;25:416. doi: 10.1186/s12871-025-03267-9 (PMC12374479; doi:10.1186/s12871-025-03267-9)

Gas Composition and Pressure in the Hypopharynx During High-Flow Oxygen Therapy Through a Nasal Cannula in Healthy Volunteers with Different Breathing Patterns (Online Data Supplement)

Andrey I. Yaroshetskiy, MD, PhD[1], Anna P. Krasnoshchekova, MD, PhD[1], Fedor D. Tkachenko[1], Alina V. Rubashchenko[1], Daniil D. Zubarev, MD[1], Vasiliy D. Konanykhin, MD[1], Maxim I. Savelenok, MD[1], Maxim M. Nosenko, MD[3], Zamira M. Merzhoeva, MD, PhD[1], and Sergey N. Avdeev, MD, PhD[1]

Affiliations

[1]Pulmonology Department, Sechenov First Moscow State Medical University (Sechenov University), Moscow, Russia

Table S1. Gas composition, pressure in the hypopharynx, and respiratory parameters at different preset flows, 40% inspiratory fraction of oxygen, and various breathing patterns of volunteers

Variable	Breathing Pattern (BP)	HFNC flow rate			p between preset flow
		30 L/min	60 L/min	80 L/min	
FiO_2, %	CM	37.1 (35.5-38.3)	40.2 (40.0-40.5)*	40.8 (40.0-41.7)**	**<0.001**
	OM	34.2 (32.0-37.0)†	38.9 (26.9-39.8)†	40.0 (27.0-40.5)†**§	**<0.001**
	HCM	34.3 (31.2-36.2) ‡	39.8 (38.5-40.3)*	40.6 (39.3-41.1)**	**<0.001**
	HOM	26.3 (22.7-29.7)#	31.7 (25.4-35.2)#*	34.1 (25.6-38.6)#**§	**<0.001**
	p between all BP	**<0.001**	**<0.001**	**<0.001**	
$FeCO_2$, %	CM	5.0 (4.1-5.4)	2.9 (2.4-3.7)*	2.4 (2.2-3.7)**	**<0.001**
	OM	4.0 (3.1-4.4)†	2.6 (2.1-3.0)†*	2.2 (2.0-2.8)†**	**<0.001**

	**HCM**	3.3 (2.9-3.9) ‡	2.5 (2.2-2.6) ‡*	2.1 (1.8-2.4) ‡**	**<0.001**
	**HOM**	2.4 (2.2-2.9)#	1.8 (1.6-2.1)#*	1.7 (1.4-2.0)#**§	**<0.001**
	**p between all BP**	**<0.001**	**<0.001**	**<0.001**	
**Expiratory pressure, cmH_2O**	**CM**	3.4 (2.9-5.3)	9.0 (6.4-10.8)*	10.5 (8.4-12.9)**§	**<0.001**
	**OM**	1.2 (0.7-1.4)†	1.6 (1.3-1.9)†	2.3 (1.4-3.0)†**	0.002
	**HCM**	4.8 (3.7-6.1)	9.9 (7.2-13.2)*	10.9 (8.4-14.5)**	**<0.001**
	**HOM**	1.2 (0.7-1.9)#	1.8 (0.7-2.4)#*	2.7 (1.6-3.2)#§	**<0.001**
	**p between all BP**	**<0.001**	**<0.001**	**<0.001**	
**Delta pressure, cmH_2O**	**CM**	3.6 (1.9-4.8)	7.0 (2.4-9.8)*	5.2 (1.2-10.4)**	**0.036**
	**OM**	1.0 (0.8-1.3)†	1.4 (0.8-1.8)†	1.1 (0.9-2.6)†	0.161
	**HCM**	6.7 (4.9-10.5) ‡	8.9 (5.2-12.9) ‡	9.7 (7.0-19.8) ‡**	**0.023**
	**HOM**	2.0 (0.9-2.8)#	1.7 (0.7-2.9)#	2.3 (1.6-4.7)#	0.465
	**p between all BP**	**<0.001**	**<0.001**	**<0.001**	
**Minimal inspiratory pressure, cmH_2O**	**CM**	1.1 (-0.5-1.5)	2.2 (0.4-4.8)	4.5 (0.8-8.5)**§	**0.023**
	**OM**	0.3 (-0.6-0.5)	0.3 (-0.0-0.7)†	0.0 (-0.6-2.0)†	0.684
	**HCM**	-3.3 (-5.6-0.3) ‡	1.0 (-1.8-1.9) ‡*	1.3 (-4.8-2.2)‡**	**0.001**
	**HOM**	-0.7 (-1.6-(-0.3))	0.1 (-0.2-0.2)#	0.1 (-2.0-1.0)	0.430
	**p between all BP**	**<0.001**	**0.004**	**0.001**	
**Exhaled tidal volume, mL**	**CM**	564.9 (480.0-825.6)	1027.0 (878.9-1273.9*	1066.5 (948.0-1422.0)**	**<0.001**
	**HCM**	830.0 (651.8-967.8) ‡	1125.8 (829.5-1323.3)*	1185.0 (948.0-1461.5)**	**<0.001**
	**p between BP**	0.002	0.491	0.108	

**Respiratory rate, min^{-1}**	**CM**	11.5 (9.3-14.0)	12.0 (8.3-16.0)	10.0 (8.0-14.0)	0.835
	**OM**	12.0 (9.3-15.8)	15.5 (10.5-16.8)	12.0 (9.0-17.0)	0.252
	**HCM**	26.5 (25.3-30.0)‡	28.0 (26.0-31.8)‡	30.0 (25.0-33.0)‡	0.412
	**HOM**	29.0 (23.5-32.0)	28.5 (28.0-32.8)	31.0 (26.0-33.0)	0.449
	**p between BP**	**<0.001**	**<0.001**	**<0.001**	
**Ventilatory ratio (modified), AU**	**CM**	0.89 (0.57-1.10)	0.84 (0.66-1.18)	0.80 (0.52-1.27)	0.692
	**HCM**	1.91 (1.54-2.18)‡	1.72 (1.09-2.04)‡	1.42 (1.01-2.33)‡	0.532
	**p between BP**	**<0.001**	**<0.001**	**<0.001**	
**Expiratory time, s**	**CM**	3.3 (2.9-4.2)	3.1 (2.7-4.0)	3.7 (2.6-6.1)	0.320
	**HCM**	1.2 (1.0-1.5)‡	1.1 (1.0-1.3)‡	1.2 (0.8-1.3)‡	0.865
	**p between all BP**	**<0.001**	**<0.001**	**<0.001**	
**Inspiratory time, s**	**CM**	2.0 (1.6-2.1)	1.7 (1.2-2.1)	1.7 (1.3-2.0)	0.267
	**HCM**	1.0 (0.8-1.1)‡	1.1 (0.9-1.2)‡	0.9 (0.8-1.0)‡	0.288
	**p between all BP**	**<0.001**	**<0.001**	**<0.001**	
**Ti/Te, ratio**	**CM**	0.50 (0.36-0.63)	0.53 (0.40-0.61)	0.34 (0.29-0.58)	0.411
	**HCM**	0.88 (0.67-1.00)	0.84 (0.78-1.00)	0.84 (0.72-1.00)	0.831
	**p between all BP**	**0.003**	**<0.001**	**0.005**	
**Visual-analog scale of comfort, points**	**CM**	8.0 (7.0-9.0)	7.0 (6.0-8.0)*	6.0 (5.0-9.0)**	**0.004**
	**OM**	8.0 (9.0-9.8)†	7.5 (6.0-8.0)*	7.0 (5.0-9.0)**	**<0.001**
	**HCM**	8.0 (7.0-9.0)	7.0 (6.0-7.8)*	6.0 (5.0-7.0)**	**0.001**
	**HOM**	8.0 (8.0-9.0)	7.0 (6.0-8.0)	6.0 (5.0-8.0)**	**0.001**

	**p between all BP**	**0.001**	**0.033**	0.323	
**Mean inspiratory flow, L/min**	**CM**	18.4 (15.4-28.4)	39.5 (29.3-45.4)*	48.4 (30.6-59.9)**	**0.001**
	**HCM**	47.4 (40.9-60.3)‡	62.5 (53.3-71.1)‡	69.3 (56.7-102.5)‡**§	**0.001**
	**p between all BP**	**0.001**	**<0.001**	**0.005**	
**Preset flow/mean inspiratory flow**	**CM**	1.63 (1.05-1.95)	1.52 (1.32-2.04)	1.65 (1.34-2.62)	0.311
	**HCM**	0.63 (0.50-0.73)	0.96 (0.84-1.13)*	1.15 (0.78-1.41)**	**<0.001**
	**p between all BP**	**0.001**	**<0.001**	**0.005**	

Data are presented as median (interquartile range). HFNC, high flow oxygen through nasal cannula; BP, breathing pattern; CM, quiet breathing with closed mouth; OM, quiet breathing with opened mouth; HCM, hyperpnea with closed mouth; HOM - hyperpnea with opened mouth; FiO_2 - measured inspiratory oxygen fraction; FeO_2 - measured expiratory oxygen fraction, $FeCO_2$ - end-expiratory carbon dioxide fraction; $FiCO_2$ - inspiratory carbon dioxide fraction; AU - arbitrary units, VAS - visual-analog scale (10 points - maximal comfort, 1 point - maximal discomfort); Te - expiratory time; Ti - inspiratory time. Significant differences (Friedman test with Bonferroni correction for multiple comparisons, $p < 0.05$) between preset flows with stable respiratory pattern: * 30 vs 60 l/min, ** 30 vs 80 l/min, § 60 vs 80 l/min. Significant differences (Friedman test with Bonferroni correction for multiple comparisons, $p < 0.05$) between different respiratory patterns with a constant preset flow: † - CM vs OM, ‡ CM vs HCM, # HCM vs HOM. Significant differences in p values are highlighted in bold.

Table S2. Gas composition, pressure in the hypopharynx, and respiratory parameters at different preset flows, 60% inspiratory fraction of oxygen, and various breathing patterns of volunteers

Variable	Breathing Pattern (BP)	HFNC flow rate			p between preset flow
		30 l/min	60 l/min	80 l/min	
FiO_2, %	CM	58.6 (57.9-60.2)	60.0 (59.6-60.3)	61.3 (60.3-62.1) ** §	**<0.001**
	OM	55.4 (49.2-58.1) †	58.4 (27.3-60.0) †	60.0 (34.8-60.3) † ** §	**0.007**
	HCM	52.3 (47.2-55.5) ‡	58.3 (57.0-60.2) *	60.0 (58.9-60.6) ‡ **	**<0.001**
	HOM	37.3 (24.2-41.7) #	40.7 (26.1-51.1) #	47.2 (31.5-55.5) #**	**0.006**
	p between all BP	**<0.001**	**<0.001**	**<0.001**	
$FeCO_2$, %	CM	3.7 (3.3-4.1)	2.6 (2.3-3.3) *	2.1 (2.0-3.2) **	**<0.001**
	OM	3.4 (2.7-4.1)	2.2 (2.1-2.6) † *	2.1 (1.9-2.4) † **	**<0.001**
	HCM	3.0 (2.6-3.7) ‡	2.2 (2.0-2.5) ‡ *	2.1 (1.7-2.3) ‡ **	**<0.001**
	HOM	2.1 (2.0-2.5) #	1.7 (1.6-2.2) # *	1.7 (1.4-1.8) #**	**<0.001**
	p between all BP	**<0.001**	**<0.001**	**<0.001**	
Expiratory pressure, cmH_2O	CM	4.0 (2.7-4.9)	8.0 (6.9-13.6) *	11.6 (8.2-13.3) **	**<0.001**
	OM	1.0 (0.8-1.3) †	2.2 (1.1-2.4) † *	2.3 (1.3-2.8) † **	**0.002**
	HCM	4.7 (3.5-5.8)	9.1 (7.2-13.8) *	11.8 (10.1-14.1) **	**<0.001**
	HOM	1.4 (1.0-1.7) #	2.0 (1.2-2.5) #	3.2 (1.4-3.9) # **	**0.004**
	p between all BP	**<0.001**	**<0.001**	**<0.001**	
Delta pressure, cmH_2O	CM	4.0 (2.6-4.7)	5.2 (4.2-10.0)	4.3 (3.0-9.6)	0.063
	OM	0.8 (0.4-1.0) †	1.3 (0.8-2.9) † *	1.5 (0.9-2.2) † **	**0.040**

	**HCM**	6.3 (3.2-8.7)	10.6 (5.7-12.3)	10.4 (8.4-16.8) ‡	0.092
	**HOM**	1.4 (0.8-3.5) #	2.3 (0.9-3.5) #	1.4 (0.8-4.7) #	0.943
	**p between all BP**	**<0.001**	**<0.001**	**<0.001**	
**Minimal inspiratory pressure, cmH_2O**	**CM**	1.0 (-1,4-1.9)	3.1 (1.6-3.9) *	4.3 (3.1-8.6) **	**<0.001**
	**OM**	0.3 (0.1-0.6)	0.3 (0.1-0.7) †	0.4 (0.2-1.0) †	0.295
	**HCM**	-0.5 (-3.2-0.3) ‡	0.3 (-1.1-1.4) ‡ *	1.3 (-0.8-3.3) ‡ **	**0.018**
	**HOM**	-0.3 (-1.8-0.3)	0.0(-1.8-0.7)	0.6 (-0.5-0.9)	0.269
	**p between all BP**	**0.011**	**<0.001**	**<0.001**	
**Exhaled tidal volume, mL**	**CM**	810.0 (675.0-1050.0)	1425.0 (1200.0-1650.0) *	1550.0 (1255.0-2200.0)**	**<0.001**
	**HCM**	1000.0 (820.0-1150.0)	1375.0 (1000.0-1625.0)*	1300.0 (1200.0-1850.0)**	**<0.001**
	**p between BP**	0.108	0.157	0.225	
**Respiratory rate, min^{-1}**	**CM**	10.5 (9.0-12.5)	10.0 (8.0-12.0)	10.0 (7.0-14.5)	0.602
	**OM**	14.5 (11.5-17.5)	16.0 (12.0-19.0)	14.0 (12.0-19.5)	0.338
	**HCM**	28.0 (25.0-30.0) ‡	26.5 (23.5-30.0) ‡	29.0 (28.0-32.0) ‡	0.118
	**HOM**	28.5 (26.5-31.0)	31.0 (27.5-33.0)	30.0 (28.0-33.0)	0.241
	**p between BP**	**<0.001**	**<0.001**	**<0.001**	
**Ventilatory ratio (modified), AU**	**CM**	0.68 (0.56-0.98)	0.76 (0.58-0.95)	0.67 (0.58-0.94)	0.692
	**OM**	0.60 (0.46-0.94) †	0.57 (0.44-0.75) †	0.53 (0.46-0.89)	0.949
	**HCM**	0.59 (0.43-0.89) ‡	1.37 (1.08-1.72) ‡ *	1.44 (1.02-1.81) † **	**<0.001**
	**HOM**	0.99 (0.72-1.14) #	1.04 (0.65-1.41) #	1.10 (0.76-1.37) #	0.229

	**p between BP**	**<0.001**	**<0.001**	**<0.001**	
**Expiratory time, s**	**CM**	3.7 (2.6-4.1)	4.1 (3.1-5.4)	4.3 (3.5-6.3) **	**0.018**
	**HCM**	1.2 (1.0-1.7) ‡	1.2 (0.9-1.5) ‡	1.2 (0.9-1.3) ‡	0.068
	**p between all BP**	**<0.001**	**<0.001**	**<0.001**	
**Inspiratory time, s**	**CM**	1.8 (1.5-2.0)	1.9 (1.8-2.0)	1.8 (1.2-2.0)	0.137
	**HCM**	0.9 (0.8-1.1) ‡	0.9 (0.9-1.1) ‡	0.9 (0.8-1.0) ‡	0.983
	**p between all BP**	**<0.001**	**<0.001**	**<0.001**	
**Ti/Te, ratio**	**CM**	0.5 (0.4-0.6)	0.4 (0.4-0.6)	0.4 (0.3-0.4) **	**0.011**
	**HCM**	0.8 (0.6-0.9) ‡	0.8 (0.6-1.0) ‡	0.8 (0.7-1.0) ‡	0.379
	**p between all BP**	**0.005**	**<0.001**	**<0.001**	
**Visual-analog scale of comfort, points**	**CM**	8.0 (8.0-9.5)	7.0 (6.0-8.0)	7.0 (6.0-8.0) **	**0.001**
	**OM**	8.5 (8.0-9.5)	7.0 (6.0-8.5) *	7.0 (5.0-8.0) **	**<0.001**
	**HCM**	8.0 (7.0-9.0)	7.0 (6.0-8.0) *	6.0 (4.5-7.0) ‡ **	**<0.001**
	**HOM**	8.0 (7.0-9.0)	7.0 (5.0-8.0) *	6.0 (5.0-7.0) **	**<0.001**
	**p between all BP**	**0.011**	**0.020**	**<0.001**	
**Mean inspiratory flow, L/min**	**CM**	23.7 (19.4-27.9)	34.4 (31.6-40.0) *	46.6 (42.1-61.6) **	**<0.001**
	**HCM**	47.4 (41.5-58.6) ‡	62.5 (54.8-70.0) ‡ *	70.2 (56.5-94.8) ‡ **	**<0.001**
	**p between all BP**	**<0.001**	**<0.001**	**0.005**	
**Preset flow/mean inspiratory flow**	**CM**	1.3 (1.1-1.6)	1.7 (1.5-2.0)	1.7 (1.3-2.0)	0.115
	**HCM**	0.6 (0.5-0.7) ‡	1.0 (0.8-1.1) ‡ *	1.1 (0.8-1.4) ‡ **	**<0.001**
	**p between all BP**	**<0.001**	**<0.001**	**0.005**	

Data are presented as median (interquartile range). HFNC, high flow oxygen through nasal cannula; BP, breathing pattern; CM, quiet breathing with closed mouth; OM, quiet breathing with opened mouth; HCM, hyperpnea with closed mouth; HOM - hyperpnea with opened mouth; FiO_2 - measured inspiratory oxygen fraction; FeO_2 - measured expiratory oxygen fraction, $FeCO_2$ - end-expiratory carbon dioxide fraction; $FiCO_2$ - inspiratory carbon dioxide fraction; AU - arbitrary units, VAS - visual-analog scale (10 points - maximal comfort, 1 point - maximal discomfort); Te - expiratory time; Ti - inspiratory time. Significant differences (Friedman test with Bonferroni correction for multiple comparisons, $p< 0.05$) between preset flows with stable respiratory pattern: * 30 vs 60 l/min, ** 30 vs 80 l/min, § 60 vs 80 l/min. Significant differences (Friedman test with Bonferroni correction for multiple comparisons, $p< 0.05$) between different respiratory patterns with a constant preset flow: † - CM vs OM, ‡ CM vs HCM, # HCM vs HOM. Significant differences in p values are highlighted in bold.

Table S3. Gas composition, pressure in the hypopharynx, and respiratory parameters at different preset flows, 80% inspiratory fraction of oxygen, and various breathing patterns of volunteers

Variable	Breathing Pattern (BP)	HFNC flow rate			p between preset flow
		30 l/min	60 l/min	80 l/min	
**FiO_2, %**	**CM**	80.2 (78.4-82.1)	82.1 (79.4-82.3)	82.1 (81.0-83.4)	0.339
	**OM**	69.8 (45.6-74.3) †	75.7 (35.7-80.1) †	76.3 (35.7-80.2) †	0.331
	**HCM**	70.2 (62.6-74.1) ‡	78.2 (75.5-80.0) ‡*	80.2 (78.1-82.3) ** §	**<0.001**
	**HOM**	42.6 (26.7-59.6) #	55.3 (30.1-65.3) #*	62.1 (35.8-73.2) #** §	**<0.001**
	**p between all BP**	**<0.001**	**<0.001**	**<0.001**	
**$FeCO_2$, %**	**CM**	3.3 (2.8-3.9)	2.7 (2.3-3.3) *	2.3 (1.9-3.1) **	**<0.001**
	**OM**	3.1 (2.4-3.5) †	2.3 (1.9-3.0) † *	2.1 (1.9-2.7) **	**0.002**
	**HCM**	2.7 (2.2-3.2) ‡	2.3 (2.0-2.9) ‡ *	1.8 (1.5-2.3) ‡ **	**<0.001**
	**HOM**	2.0 (1.8-2.3) #	1.8 (1.4-2.0) #*	1.5 (1.3-1.8) **	**<0.001**
	**p between all BP**	**<0.001**	**<0.001**	**<0.001**	
**Expiratory pressure, cmH_2O**	**CM**	4.3 (3.4-6.1)	7.7 (6.4-12.2)*	10.6 (8.0-14.0)**	**<0.001**
	**OM**	1.2 (0.7-1.6) †	1.3 (0.8-1.7) †	1.7 (0.6-3.6) †	0.101
	**HCM**	4.9 (4.1-7.5)	8.8 (6.6-12.4)*	11.9 (11.1-15.9)** §	**<0.001**
	**HOM**	1.5 (1.0-2.2) #	1.6 (0.9-2.5) #	2.4 (0.9-3.6) #	0.199
	**p between all BP**	**<0.001**	**<0.001**	**<0.001**	
**Delta pressure, cmH_2O**	**CM**	4.2 (2.2-5.3)	4.1 (2.8-8.8)	4.9 (3.0-11.3)	0.199
	**OM**	0.9 (0.5-1.5) †	1.2 (0.5-1.5) †	1.3 (0.5-2.4) †	0.787

	**HCM**	6.2 (4.7-9.3)	8.0 (6.0-13.2) ‡	10.7 (9.4-15.4) ‡	0.149
	**HOM**	2.0 (0.7-3.5) #	1.5 (0.6-3.0) #	2.3 (1.0-4.5) #	0.689
	**p between all BP**	**<0.001**	**<0.001**	**<0.001**	
**Minimal inspiratory pressure, cmH_2O**	**CM**	0.8 (0.3-1.5)	3.2 (1.8-5.0)*	4.7 (3.1-7.9)**	**<0.001**
	**OM**	0.3 (-0.1-0.5)	0.2 (0.0-0.4)†	0.5 (0.1-1.2)†	0.255
	**HCM**	-1.3 (-2.7- -0.1) ‡	0.5 (-0.5-1.9) ‡	1.1 (0.3-2.9) ‡	**0.002**
	**HOM**	-0.2 (-1.9-0.4)	0.1(-0.3-0.3)*	0.0 (-1.7-0.6) #**	0.980
	**p between all BP**	**<0.001**	**<0.001**	**<0.001**	
**Exhaled tidal volume, mL**	**CM**	855 (695.0-1175.0)	1225.0 (1000.0-1700.0)*	1300.0 (1100.0-1800.0)**	**<0.001**
	**HCM**	920.0 (850.0-1087.5)	1375.0 (1062.5-1600.0)*	1250.0 (1200.0-1750.0)**	**<0.001**
	**p between BP**	0.251	0.491	0.819	
**Respiratory rate, min^{-1}**	**CM**	11.5 (8.0-15.8)	12.0 (8.0-12.8)	10.0 (7.0-16.0)	0.571
	**OM**	15.5 (10.5-18.0)	15.0 (12.0-17.8)†	15.0 (12.0-18.0)	0.828
	**HCM**	30.0 (26.0-31.0) ‡	28.5 (25.0-31.0) ‡	30.0 (26.0-32.0) ‡	0.206
	**HOM**	29.0 (26.0-31.8)	32.5 (29.3-33.0)	30.0 (26.0-33.0)	0.173
	**p between BP**	**<0.001**	**<0.001**	**<0.001**	
**Ventilatory ratio (modified), AU**	**CM**	0.7 (0.4-1.0)	0.7 (0.5-1.0)	0.6 (0.4-0.8)	0.692
	**OM**	0.5 (0.4-0.8)†	0.5 (0.4-0.9)	0.5 (0.3-0.7)	0.810
	**HCM**	1.3 (1.0-1.7) ‡	1.5 (1.0-1.8) ‡	1.3 (0.9-1.8) ‡	0.128
	**HOM**	1.0 (0.8-1.3)	1.1 (0.7-1.3) #	0.9 (0.5-1.4)	0.692

	**p between BP**	**<0.001**	**<0.001**	**<0.001**	
**Expiratory time, s**	**CM**	3.7 (2.5-5.1)	3.8 (3.0-6.0)	3.6 (2.1-7.9)	0.646
	**HCM**	1.2 (1.0-1.4) ‡	1.2 (1.1-1.3) ‡	1.2 (1.0-1.3) ‡	0.426
	**p between all BP**	**<0.001**	**<0.001**	**<0.001**	
**Inspiratory time, s**	**CM**	1.5 (1.1-2.0)	1.7 (1.2-1.9)	1.6 (1.1-1.7)	0.408
	**HCM**	1.0 (0.8-1.2) ‡	1.0 (0.8-1.1) ‡	0.9 (0.8-1.0) ‡	0.093
	**p between all BP**	**<0.001**	**<0.001**	**<0.001**	
**Ti/Te, ratio**	**CM**	0.5 (0.3-0.6)	0.4 (0.3-0.6)	0.4 (0.2-0.5)	0.411
	**HCM**	0.9 (0.8-0.9) ‡	0.9 (0.8-0.9) ‡	0.8 (0.7-0.9) ‡	0.672
	**p between all BP**	**<0.0001**	**<0.0001**	**<0.0001**	
**Visual-analog scale of comfort, points**	**CM**	8.0 (8.0-9.8)	7.0 (6.0-8.0)*	6.0 (4.0-7.0)** §	**<0.001**
	**OM**	8.5 (8.0-10.0)	6.5 (5.3-8.0)*	7.0 (4.0-8.0)** §	**<0.001**
	**HCM**	8.0 (7.3-9.0)	6.5 (5.3-8.0)*	6.0 (4.0-8.0)**	**<0.001**
	**HOM**	8.0 (7.0-9.0)	6.0 (5.0-8.0)*	5.0 (4.0-7.0)** §	**<0.001**
	**p between all BP**	**<0.001**	0.215	0.112	
**Mean inspiratory flow, L/min**	**CM**	24.8 (18.3-39.3)	34.8 (27.9-45.0)*	47.4 (34.9-56.5)** §	**<0.001**
	**HCM**	42.7 (40.2-50.4) ‡	59.3 (50.4-79.0) ‡*	71.1 (60.1-89.7) ‡**	**<0.001**
	**p between all BP**	**<0.001**	**0.003**	**<0.001**	
**Preset flow/mean inspiratory flow**	**CM**	1.2 (0.8-1.6)	1.7 (1.3-2.2)*	1.7 (1.4-2.3)**	**0.006**
	**HCM**	0.7 (0.6-0.7) ‡	1.0 (0.8-1.2) ‡*	1.1 (0.9-1.3) ‡**	**<0.001**
	**p between all BP**	**<0.001**	**0.003**	**<0.001**	

Data are presented as median (interquartile range). HFNC, high flow oxygen through nasal cannula; BP, breathing pattern; CM, quiet breathing with closed mouth; OM, quiet breathing with opened mouth; HCM, hyperpnea with closed mouth; HOM - hyperpnea with opened mouth; FiO_2 - measured inspiratory oxygen fraction; FeO_2 - measured expiratory oxygen fraction, $FeCO_2$ - end-expiratory carbon dioxide fraction; $FiCO_2$ - inspiratory carbon dioxide fraction; AU - arbitrary units, VAS - visual-analog scale (10 points - maximal comfort, 1 point - maximal discomfort); Te - expiratory time; Ti - inspiratory time. Significant differences (Friedman test with Bonferroni correction for multiple comparisons, $p < 0.05$) between preset flows with stable respiratory pattern: * 30 vs 60 l/min, ** 30 vs 80 l/min, § 60 vs 80 l/min. Significant differences (Friedman test with Bonferroni correction for multiple comparisons, $p < 0.05$) between different respiratory patterns with a constant preset flow: † - CM vs OM, ‡ CM vs HCM, # HCM vs HOM. Significant differences in p values are highlighted in bold.

Table S4. Influence of preset inspiratory oxygen fraction at constant flow rates on different respiratory parameters during quiet breathing wilt closed mouth (p-values)

Variable	HFNC flow rate		
	30 L/min	60 L/min	80 L/min
**$FeCO_2$, %**	**<0.001**	**0.010**	**<0.001**
**FiO_2, %**	**<0.001**	**<0.001**	**<0.001**
**Expiratory pressure, cmH_2O**	NS	NS	NS
**Minimal inspiratory pressure, cmH_2O**	NS	NS	NS
**Delta pressure, cmH_2O**	NS	NS	NS
**Exhaled tidal volume, mL**	NS	NS	NS
**Respiratory rate, min^{-1}**	NS	NS	NS
**Ventilatory ratio (modified), AU**	**0.014**	**0.022**	**<0.001**
**Expiratory time, s**	NS	NS	NS
**Inspiratory time, s**	NS	NS	NS
**Ti/Te, ratio**	NS	NS	NS
**Visual-analog scale of comfort, points**	NS	NS	**<0.001**
**Mean inspiratory flow, L/min**	NS	NS	NS

Significant differences in p values are highlighted in bold. Abbreviations: HFNC - high-flow nasal cannula, F_ECO_2 - end-expiratory carbon dioxide fraction, FiO_2 - measured inspiratory oxygen fraction, Ti - inspiratory time, Te - expiratory time, AU - arbitrary units, NS - non-significant.

Table S5. Answers about subject's comfort.

Questions and answers	n (%)
**Question #1: What was the general discomfort?**	
**Answers:**	
- Difficulty exhaling	14 (70)
- Dizziness	10 (50)
- Numbness in the arms	6 (30)
- Lung overinflation	5 (25)
- Cramped fingers	4 (20)
- Cold hands	4 (20)
- Headache	3 (15)
- Difficulty in breathing	2 (10)
- Pressure inside the chest	1 (5)
- Palpitations	0 (0)
**Question #2: What was the local discomfort?**	
**Answers:**	
- Increased secretion in the nose	11 (55)
- Blocked ears	11 (55)
- Blowing hard	9 (45)
- Stuffy nose	9 (45)
- Dry throat	6 (30)

- Noise in the ears	5 (25)
**Question #3: Which was more comfortable: open or closed mouth?**	
**Answers:**	
- Open mouth is more comfortable	14 (70)
- Closed mouth is more comfortable	6 (30)

Figure S1. Influence of preset oxygen fraction during hyperpnea through open mouth with the preset flow rate of 60 L/min on measured inspiratory oxygen fraction

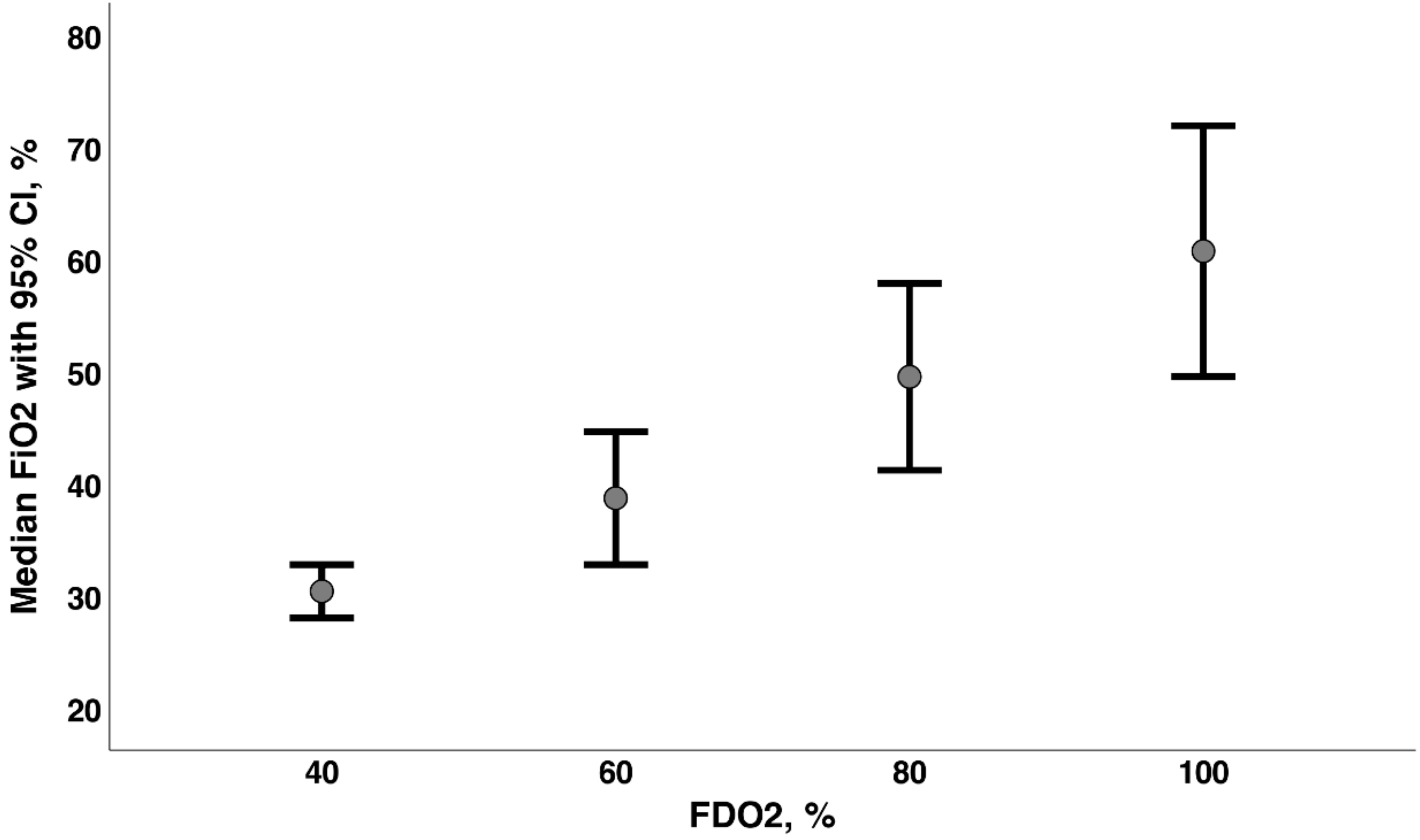

Figure S2. Influence of preset oxygen fraction during hyperpnea through open mouth with the preset flow rate of 80 L/min on measured inspiratory oxygen fraction

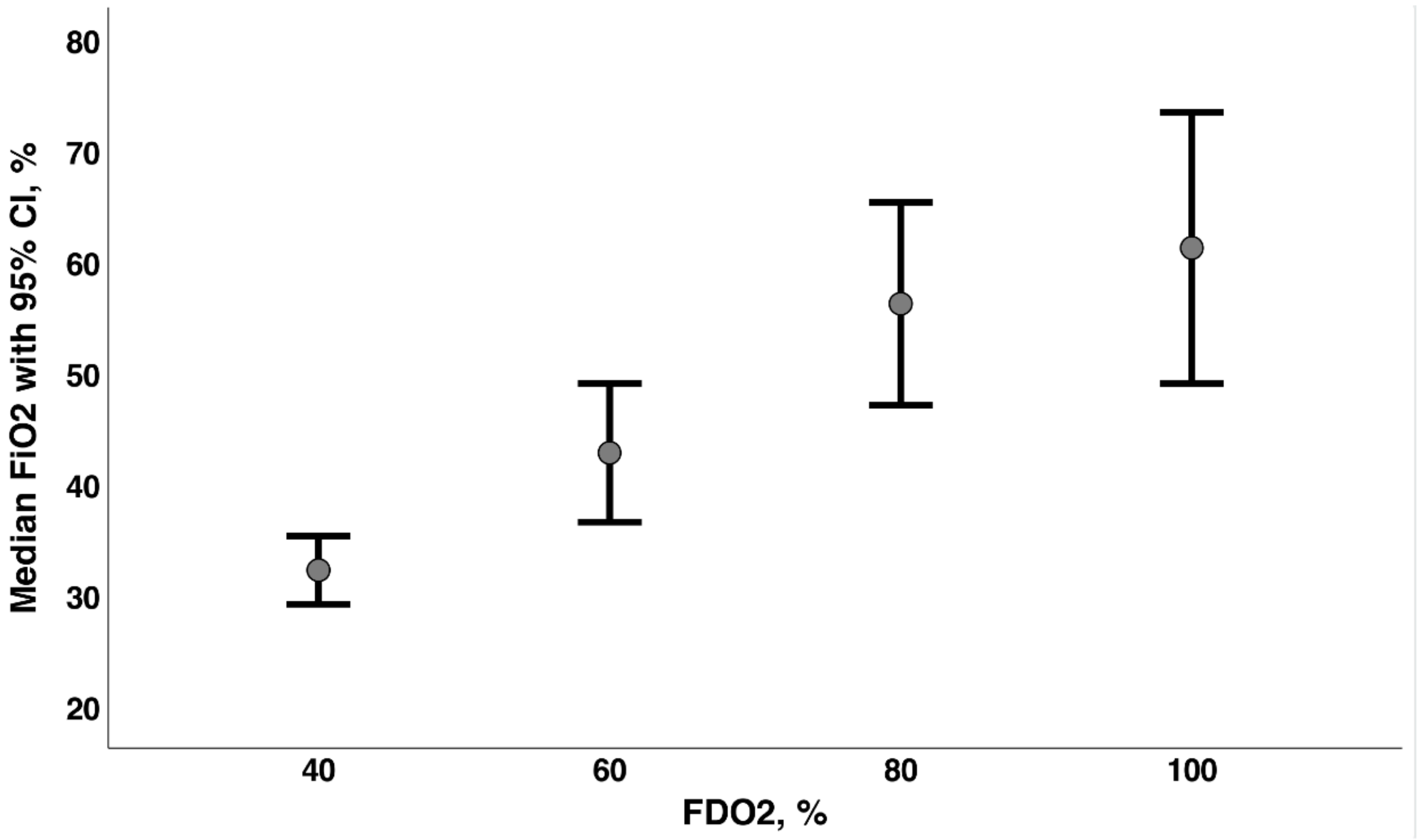

Figure S3. Influence of preset oxygen fraction with different preset flows on respiratory parameters during quiet breathing with closed mouth. *A:* Measured end-expiratory carbon dioxide fraction in the hypopharynx. *B:* Ventilatory ratio. *C:* Subject's comfort expressed as visual-analog scale. Blue boxes: preset flow rate 30 L/min. Grey boxes: preset flow rate 60 L/min. Red boxes: preset flow rate 80 L/min. Abbreviations: F_DO_2 - preset oxygen fraction, F_ECO_2 - end-expiratory carbon dioxide fraction, AU - arbitrary units, VAS - visual-analog scale (10 points - maximal comfort, 1 point - maximal discomfort).

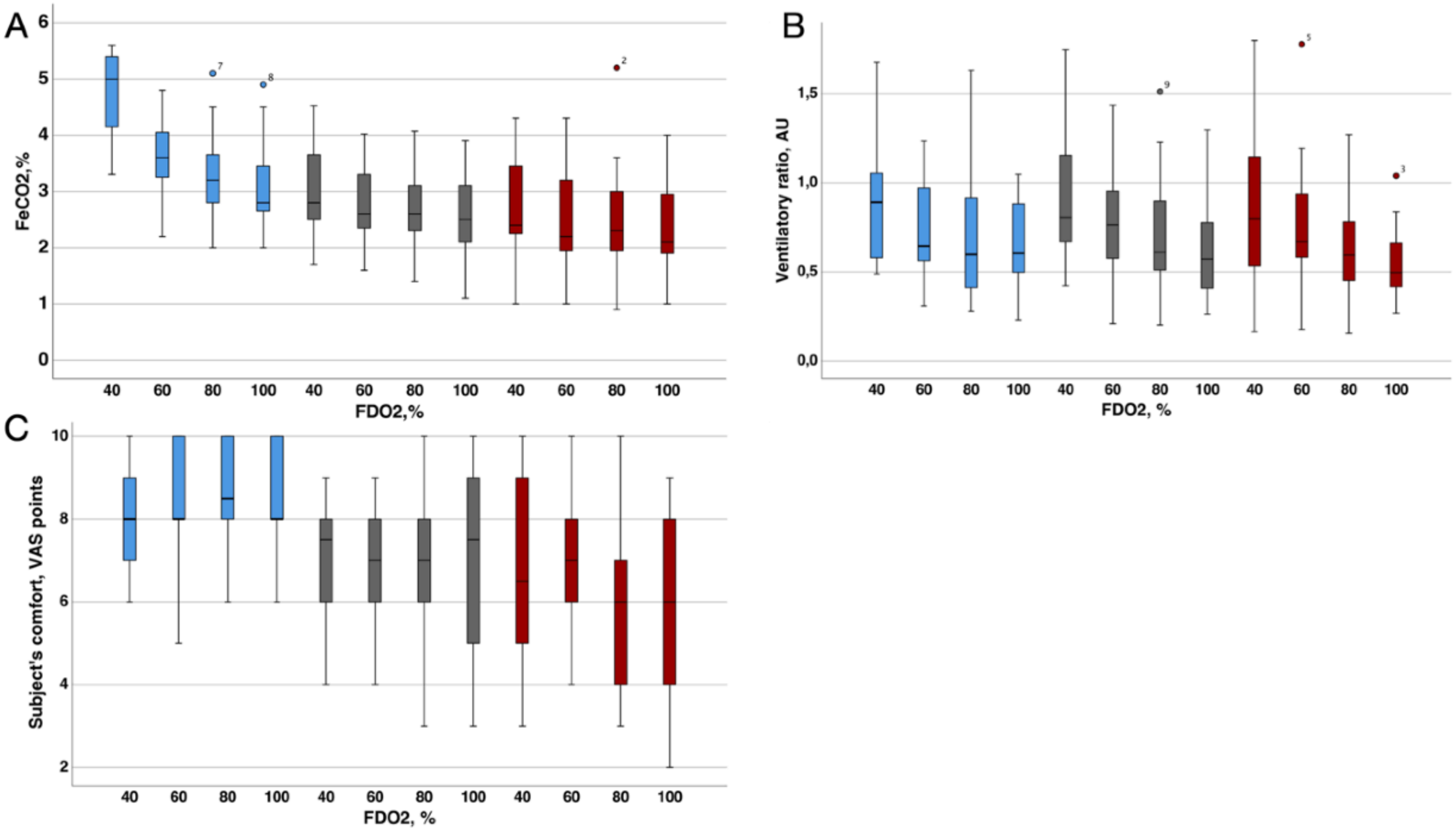

Figure S4. Influence of preset oxygen fraction and breathing pattern on measured end-expiratory carbon dioxide fraction in the hypopharynx at the preset flow rate of 30 L/min. *A:* CM - quiet breathing with closed mouth. *B:* OM - quiet breathing through open mouth. *C:* HCM - hyperpnea with closed mouth. *D:* HOM - hyperpnea through open mouth. Abbreviations: F_ECO_2 - end-expiratory carbon dioxide fraction, F_DO_2 - preset oxygen fraction.

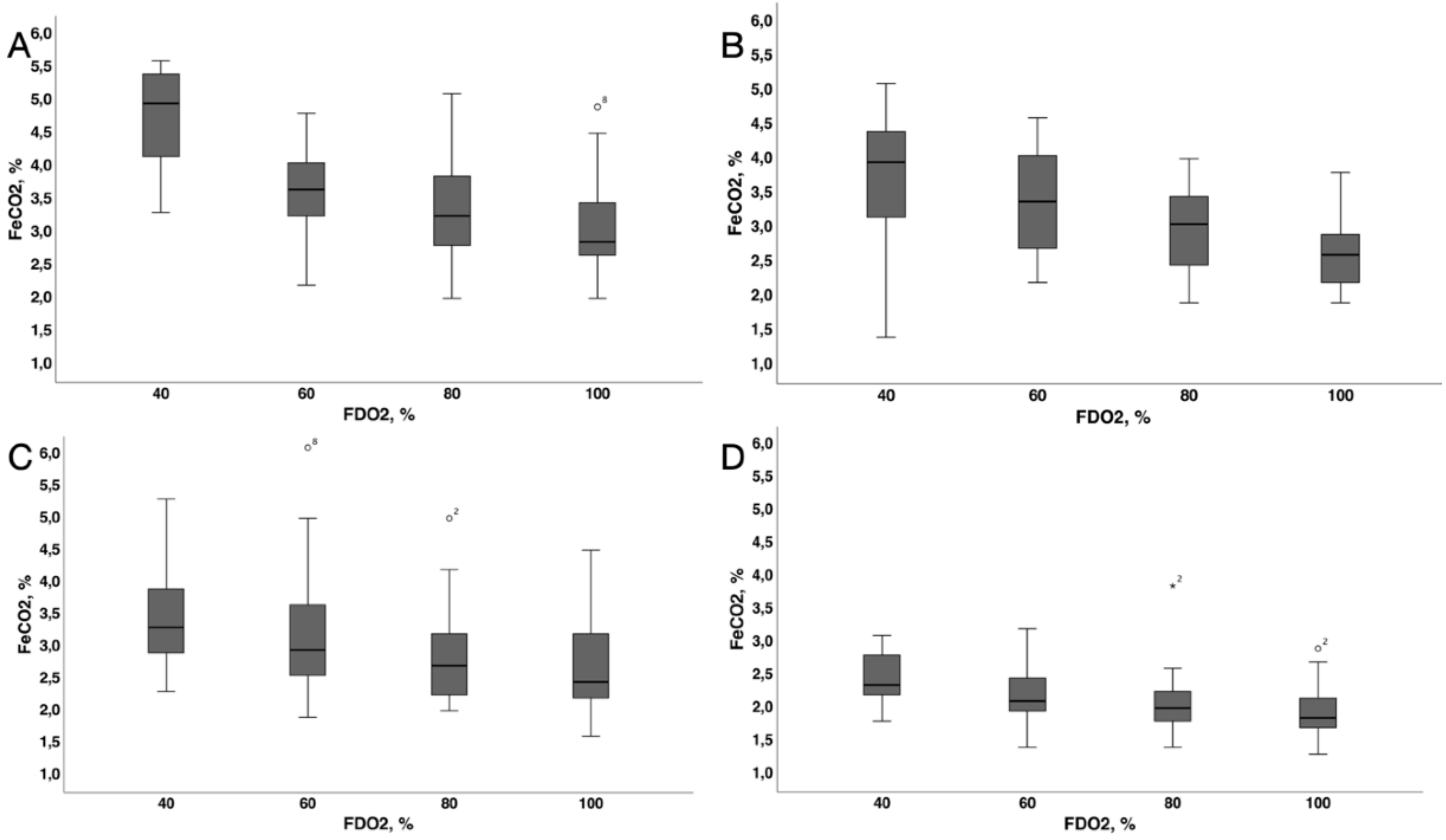

Figure S5. Influence of preset oxygen fraction and breathing pattern on the modified ventilatory ratio at preset flow rate of 30 L/min. *A:* CM - quiet breathing with closed mouth. *B:* OM - quiet breathing through open mouth. *C:* HCM - hyperpnea with closed mouth. *D:* HOM - hyperpnea through open mouth. Abbreviations: AU - arbitrary units, F_DO_2 - preset oxygen fraction.

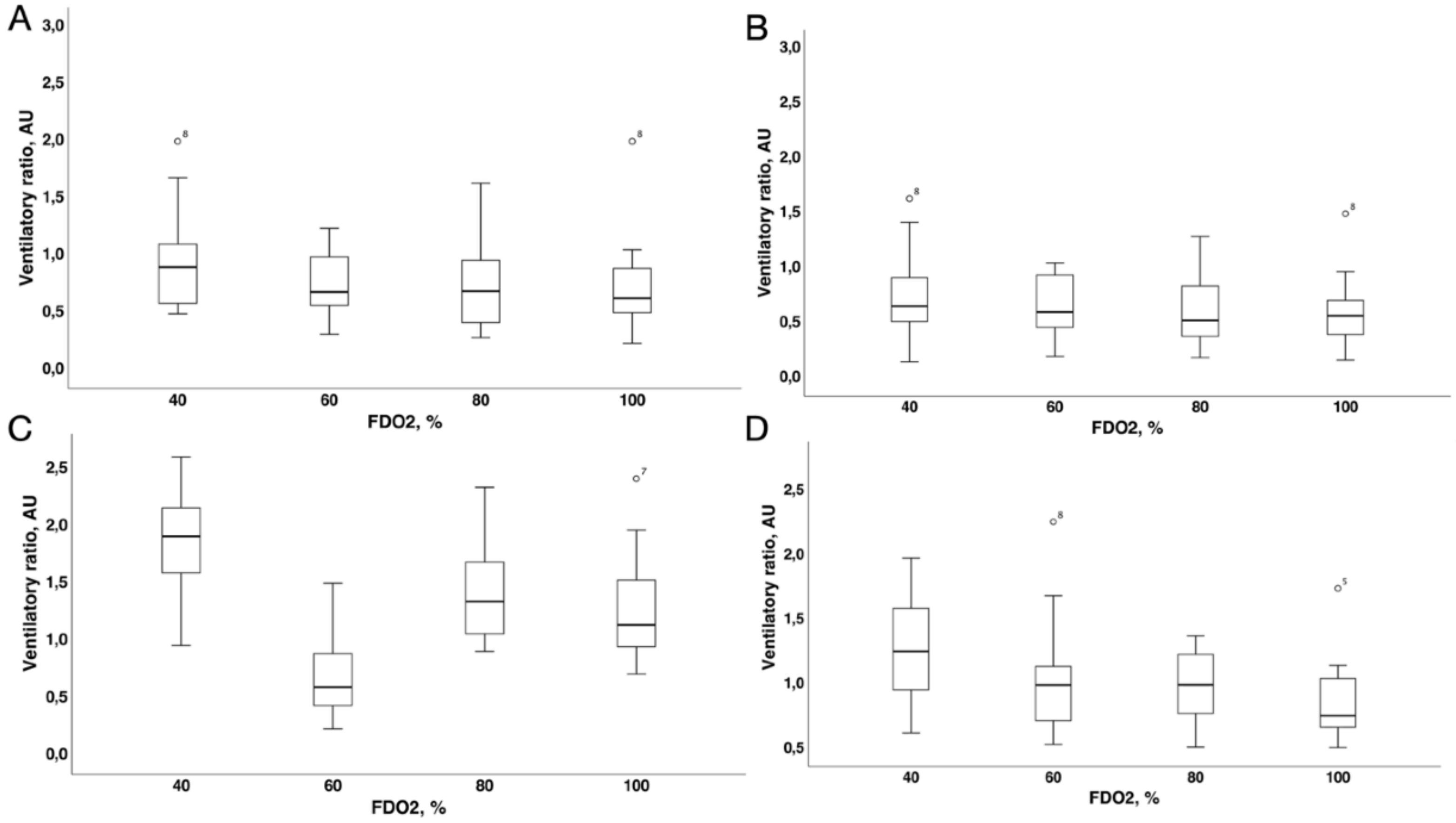

Supplement: Supplementary file 1 — Supplementary Material 1. [file 12871_2025_3267_MOESM1_ESM.pdf]
